# Multiple Myeloma-Derived Extracellular Vesicles Impair Normal Hematopoiesis by Acting on Hematopoietic Stem and Progenitor Cells

**DOI:** 10.3389/fmed.2021.793040

**Published:** 2021-12-16

**Authors:** Ilaria Laurenzana, Stefania Trino, Daniela Lamorte, Angelo De Stradis, Michele Santodirocco, Alessandro Sgambato, Luciana De Luca, Antonella Caivano

**Affiliations:** ^1^Laboratory of Preclinical and Translational Research, Centro di Riferimento Oncologico della Basilicata (IRCCS-CROB), Rionero in Vulture, Italy; ^2^Institute for Sustainable Plant Protection, National Research Council (CNR), Bari, Italy; ^3^Trasfusional Medicine Department, Puglia CBB, Casa Sollievo Della Sofferenza Hospital, San Giovanni Rotondo, Italy; ^4^Scientific Direction, Centro di Riferimento Oncologico della Basilicata (IRCCS-CROB), Rionero in Vulture, Italy; ^5^Unit of Clinical Pathology, Centro di Riferimento Oncologico della Basilicata (IRCCS-CROB), Rionero in Vulture, Italy

**Keywords:** multiple myeloma, extracellular vesicles, hematopoiesis, differentiation, hematopoietic stem and progenitor cells, microRNA, mRNA target

## Abstract

Multiple myeloma (MM) is characterized by the abnormal proliferation of clonal plasma cells (PCs) in bone marrow (BM). MM-PCs progressively occupy and likely alter BM niches where reside hematopoietic stem and progenitor cells (HSPCs) whose viability, self-renewal, proliferation, commitment, and differentiation are essential for normal hematopoiesis. Extracellular vesicles (EVs) are particles released by normal and neoplastic cells, such as MM cells. They are important cell-to-cell communicators able to modify the phenotype, genotype, and the fate of the recipient cells. Investigation of mechanisms and mediators underlying HSPC-MM-PC crosstalk is warranted to better understand the MM hematopoietic impairment and for the identification of novel therapeutic strategies against this incurable malignancy. This study is aimed to evaluate whether EVs released by MM-PCs interact with HSPCs, what effects they exert, and the underlying mechanisms involved. Therefore, we investigated the viability, cell cycle, phenotype, clonogenicity, and microRNA profile of HSPCs exposed to MM cell line-released EVs (MM-EVs). Our data showed that: (i) MM cells released a heterogeneous population of EVs; (ii) MM-EVs caused a dose-dependent reduction of HSPCs viability; (iii) MM-EVs caused a redistribution of the HSPC pool characterized by a significant increase in the frequency of stem and early precursors accompanied by a reduction of late precursor cells, such as common myeloid progenitors (CMPs), megakaryocyte erythroid progenitors (MEPs), B and NK progenitors, and a slight increase of granulocyte macrophage progenitors (GMPs); (iv) MM-EVs caused an increase of stem and early precursors in S phase with a decreased number of cells in G_0_/G_1_ phase in a dose-dependent manner; (v) MM-EVs reduced the HSPC colony formation; and (vi) MM-EVs caused an increased expression level of C-X-C motif chemokine receptor type 4 (CXCR4) and activation of miRNAs. In conclusion, MM cells through the release of EVs, by acting directly on normal HSPCs, negatively dysregulate normal hematopoiesis, and this could have important therapeutic implications.

## Introduction

Hematopoietic stem and progenitor cells (HSPCs) are responsible for the production of all blood and immune cells over the life span (1). Understanding the mechanisms and factors regulating HSPC populations is important for two reasons. First, it allows to understand blood cell production under both normal and pathological conditions; thus, it could help to decipher the steps/pathways that lead to neoplastic cell transformation (2). For example, myelodysplastic syndromes arise from a small population of disease-initiating HSCs that persist and expand despite therapies and are responsible for disease progression and relapse (3). Second, it is important for the development of new therapeutic strategies (2). For instance, identification of the role played by the Notch pathway has led to the development of strategies for the *ex vivo* expansion of HPSCs from human cord blood for allogeneic transplantation (4). In acute myeloid and lymphoblastic leukemia, several small molecule inhibitors, short peptides, and antibodies have been developed to disrupt the C-X-C motif chemokine receptor type 4 (CXCR4)-mediated interaction between leukemic stem cells and the supportive bone marrow (BM) niche that contributes to chemoresistance (5).

Multiple myeloma (MM), to date an incurable hematological malignancy, is characterized by BM infiltration by a clonal population of malignant plasma cells (MM-PCs). The release of soluble factors, namely, cytokines, chemokines, and extracellular vesicles (EVs), from the malignant PCs and neighboring cells leads to alterations of the BM milieu, which, in response, becomes supportive to the neoplastic cells. HSPCs reside in the BM niche and their viability, self-renewal, proliferation, commitment, and differentiation are essential for normal hematopoiesis. Common signaling pathways are utilized by HSPCs and MM cells to mediate their localization and proliferation. Paracrine and autocrine signals, such as the stromal cell-derived factor 1 (SDF-1)/CXCR4 and interleukin (IL-6) axis, mediate not only PC proliferation and the initiation of premalignant disease, but also BM HSPC homing, retention, proliferation, and egress. In particular, CXCR4 expression on HSPCs is necessary to keep these cells in the SDF-1-enriched BM microenvironment.

Clinical manifestations of MM at diagnosis include osteolytic bone lesions, hypercalcemia, and a suppressed hematopoietic function. It has been hypothesized that hematopoiesis compromission might be due to “an anatomic crowding out” of HSPCs by BM MM-PCs proliferation (6–8). Recently, it has been reported that the number of HSPCs in MM BM was negatively correlated with CD138^+^ cell number and that the MM BM microenvironment suppressed HSC differentiation to promote MM-associated anemia (9). In addition, Bruns et al. (6) reported that HSPCs, in particular megakaryocyte-erythroid progenitors, are diminished in the BM of patients with MM. However, the underlying mechanisms of MM hematopoietic suppression are incompletely understood.

Extracellular vesicles are one of the most important cell-to-cell communicators leading to modifications of phenotype, genotype, and the fate of the recipient cells. Notably, neoplastic EVs, such as those derived from the BM microenvironment, mediate differentiation and homing of HSPCs, transforming capacity, and even their possible therapeutic ability in the field of allogeneic transplantation (10, 11). In BM, PCs take advantage of the local cell populations, such as mesenchymal stromal cells (MSCs), osteoblasts (OBs)/osteoclasts (OCs), endothelial cells, and immune cells, and are sustained by a supportive milieu rich in cytokines, growth factors, and EVs. In particular, in MM, EVs (i) are able to promote angiogenesis (12, 13); (ii) “encourage” PC growth facilitating MM progression (14); (iii) play a pivotal role in immune modulation to promote the viability and proliferation of myeloid-derived suppressor cells (13); (iv) positively modulate pre-OC migration (15); and finally, (v) are associated with the acquisition of drug resistance (16, 17). Of note, we and others found MM-EVs in the peripheral blood of patients with MM and showed that their circulating levels and associated biomarkers are positively correlated with clinical parameters (18, 19).

To our knowledge, to date, no data are available about EV-mediated communication between MM-PCs and healthy HSPCs and the resulting effect on HSPCs. We analyzed this communication and performed a qualitative assessment of distinct HSPC subsets, evaluating cell viability and cycle, clonogenic functions, and the miRNA profile after exposure to MM-EVs.

## Materials and Methods

### Multiple Myeloma Cell Line and HSPCs

Multiple myeloma cell line, RPMI 8226, was acquired from American Type Culture Collection (Rockville, MD, USA). Cells were cultured in RPMI-1640 medium (Gibco, Life Technologies, Carlsbad, CA, USA) supplemented with 10% of fetal bovine serum (FBS; Gibco), 1% of penicillin-streptomycin (Gibco), and 2 mM of L-glutamine (Gibco) at 37°C and 5% CO_2_.

Hematopoietic stem and progenitor cells were isolated from umbilical cord blood cells (UCBs). UBCs were provided by Cord Blood Bank of Research Institute “Casa Sollievo della Sofferenza,” San Giovanni Rotondo, Italy. The Ethics Committee of IRCCS-Centro di Riferimento Oncologico della Basilicata (CROB) and that of Research Institute “Casa Sollievo della Sofferenza” approved the current study. Signed informed consents were obtained from all subjects prior to UBC collection.

To obtain CD34^+^ cells, UCBs were centrifuged at 1,578 × g for 10 min at room temperature (RT), and mononuclear cells were recovered from the buffy coat fraction. Afterward, cells were labeled with the CD34 MicroBead Kit (Miltenyi Biotec, Auburn, CA, USA) following the protocol of the manufacturer. AutoMACS Pro separator (Miltenyi Biotec) was used to purify CD34^+^ cells. The purity of isolated CD34^+^ cells was verified by the FACS CANTO II flow cytometer using DIVA software (Becton Dickinson, BD Biosciences, San Jose, CA, USA), and it ranged between 90 and 95%.

We point out that UCB bags were collected within 5 days before CD34^+^ isolation.

### Isolation of EVs From MM Cell Line

Multiple myeloma-EVs were isolated from 200 × 10^6^ RPMI 8226 cells. In particular, cells were cultured (1.2 × 10^6^ cells/ml) in RPMI-1640 without FBS for 48 h. Cells were collected and centrifuged at 300 × g for 5 min at RT. EVs were isolated as previously reported (18). Briefly, the supernatant was submitted to different centrifugation steps, and the EV pellet was washed with 0.22-μm pre-filtered phosphate buffer saline (PBS w/o calcium and magnesium, Gibco). Finally, the EV pellet was resuspended in 1,500 μl of 0.02-μm pre-filtered PBS.

### Quantification of EVs by Nanoparticle Tracking Analysis (NTA) and of EV Protein Concentration by Bicinchoninic Acid (BCA) Assay

Size distribution and concentration of MM-derived EVs were determined by NTA using a NanoSight NS300 instrument (Malvern Panalytical Instrument, UK). Samples were diluted at 1:80 in PBS w/o calcium and magnesium in a final volume of 400 μl according to the recommendations of the manufacturer. NTA instrument settings, D10, D90 values, mode, mean were reported as previously indicated (18).

Extracellular vesicle protein concentration was quantified using the BCA Protein Assay Kit (Thermo Scientific, Rockford, IL, USA). Different concentration of bovine serum albumin (BSA) was used to generate a standard curve. Five microliters of MM-derived EVs were lysed in a cold lysis buffer (100 mM Tris-HCl pH 7.5, 600 mM NaCl, 4% Triton X-100, 0.4% sodium dodecyl sulfate [SDS], 20 mM ethylenediaminetetraacetic acid [EDTA]) for 30 min on ice. Five microliters of sample lysate were used for the assay following the instructions of the manufacturer. Samples were incubated at 37°C for 30 min. Then, the absorbance at 560 nm was measured using the VICTOR Nivo (Perkin Elmer, Waltham, MA, USA), and the protein concentration was determined based on the standard curve.

### Analysis of EV Quality and Associated Surface Markers by Trasmission Electron Mi Croscopy (TEM) and Flow Cytometry

Twenty microliters of EV sample suspension were applied to a Pioloform-coated Nickel grid (200 mesh; TAAB Laboratories Equipment Ltd., Aldermaston, UK). The grid was floated for 2 min on the sample drop and rinsed on a 20 μl double distilled water drop. Negative staining was performed with 200 μl of 2% w/v uranyl acetate solution (TAAB Laboratories Equipment Ltd.). After draining off the excess staining solution, the specimen was examined in a Philips Morgagni 282D TEM, operating at 60 kV. Electron micrographs of negatively stained samples were photographed on Kodak electron microscope film 4489 (Kodak Company, Rochester, NY, USA).

Multiple myeloma-associated markers were evaluated on MM-derived EVs using the FACS CANTO II flow cytometer and DIVA software (BD Biosciences). Flow cytometer setting was previously reported (18).

### Treatment of HPSCs With MM-EVs

Immediately after isolation, CD34^+^ cells were cultured in StemMACS HSC Expansion Media XF, supplemented with StemMACS HSC Expansion Cocktail (Miltenyi Biotec). Specifically, 8.5 × 10^5^ cells in 1 ml were treated with 200 and 400 μg of MM-EVs and with 0.02 μm filtered PBS (as control) and were incubated at 37°C and 5% CO_2_ for 20 h. CD34^+^ cells were then centrifuged at 500 × g, washed with PBS, and used for subsequent functional analysis.

Of note, we cannot work with frozen CD34^+^ cells since we previously observed alteration in the distribution of early and late progenitors post-thaw HSPCs compared with the fresh ones.

### Cell Count

Viable CD34^+^ cells were counted in a Burker chamber using Trypan Blue staining.

### Analysis of Differentiation Markers on HSPCs

Fifty thousand EV-treated and untreated CD34^+^ cells were labeled with fluorescein isothiocyanate (FITC) anti-CD34 (clone 8G12; BD), PE-Cyanine 7 (PE-Cy7) anti-CD38 (clone HB-7; BD), APC-H7 anti-CD45RA (cloneHI100; BD Pharmingen), APC anti-CD10 (clone HI10a, BD), peridinin chlorophyll Cyanine 5.5 (PerCP-Cy5.5) anti-CD90 (Clone 5E10; BD Pharmingen), and PE anti- CD123 (clone7G3; BD Pharmingen) monoclonal conjugated antibodies, at RT for 15 min in the dark. After incubation, cells were washed and resuspended in PBS. Then, 10,000 events were acquired on the FACS CANTO II flow cytometer and analyzed by DIVA software (BD).

### Cell Cycle Analysis

CD34^+^ cells were labeled with FITC anti-CD34 (BD) and PE-Cy7 anti-CD38 (BD) conjugated monoclonal antibodies at RT for 15 min in the dark. After incubation, cells were washed and resuspended in 200 μl of Dulbecco's Modified Eagle's Medium (DMEM; Gibco). Subsequently, Vybrant DyeCycle Violet (Invitrogen Molecular Probes, Eugene, OR, USA) was added to a final concentration of 5 μM, and cells were incubated at 37°C for 30 min in the dark. Samples were then acquired on FACS CANTO II, and the cell cycle was analyzed by Kaluza 2.0 software (Beckman Coulter, Life Sciences, Indianapolis, IN, USA).

### Colony-Forming Unit (CFU) Assay

CD34^+^ cells (1.5 × 10^3^ cells), treated or not with EVs for 20 h, were plated in two 35-mm dishes in MethoCult Classic H4434 (Stem Cell Technologies, Vancouver, BC, Canada) and incubated at 37°C in a humidified atmosphere at 5% CO_2_. MethoCult classic medium H4434 already contains SCF, IL3, GM-CSF, EPO, and FBS. After another 14 days, colonies were counted with an inverted microscope (Zeiss, Germany). The absolute number of colonies, as a sum of the two dishes, was calculated. The percentage of specific colonies was calculated on the total number of colonies for each experimental condition.

### Expression of CXCR4 on HSPCs

The expression of CXCR4 was evaluated on CD34^+^ cells treated or not with EVs after 20 h. Cells were incubated with FITC anti-CD34 (BD), PE-Cy7 anti-CD38 (BD), and APC anti-CXCR4 (clone 12G5; BD Pharmingen) conjugated monoclonal antibodies at RT for 15 min in the dark. After incubation, cells were washed and suspended in PBS. Then, 10,000 events were acquired on FACS CANTO II and analyzed by DIVA software (BD).

### Digital PCR for miR-21, miR-34, miR-150, and miR-155 in HSPCs and EVS and Identification of miRNA Targets

Droplet digital PCR was performed for the measurement of miRNAs 21-5p, 34a-5p, 150-5p, and 155-5p in MM-EVs and HSPCs.

Total RNA was extracted from of MM-EVs with Trizol reagent (Life Technologies, Carlsbad, CA, USA) according to the instructions of the manufacturer. Briefly, Trizol was added to 300 μl of MM-EVs. Subsequently, RNA was resuspended in 70 μl elution buffer and stored at −80°C until use.

RNA from about 3 × 10^5^ HSPCs, treated or not with EVs, was extracted using RNA/DNA/PROTEIN Purification Plus Micro Kit (NorgenBiotek Corporation, Canada).

RNA from EVs and cells was quantified using the Nanodrop Spectrophotometer (Thermo Scientific, Wilmington, DE, USA).

To quantify miRNAs in HSPCs, 2.5 ng of RNA from HSPCs were reversely transcribed by a Veriti 96-well thermal cycler (Thermo Fisher Scientific, Applied Biosystems, Foster City, CA, USA) using TaqMan™ Advanced miRNA cDNA Synthesis Kit and miRNA-specific stem-loop primers (Cat. A28007, Applied BioSystems). Twenty microliters of the reaction mixture containing 5 μl of cDNA solution, 10 μl of digital PCR™ Supermix (Bio-Rad, Hercules, CA, USA), and 1 μl of Taqman probe mix and DEPC H_2_O were loaded into a plastic cartridge (Bio-Rad) with 70 μl of QX100 Droplet Generation oil (Bio-Rad) and then placed into the QX100 Droplet Generator (Bio-Rad). The droplets generated from each sample were transferred to a 96-well PCR plate (Eppendorf, Germany). PCR amplification was carried on a Veriti 96-well thermal cycler (Applied Biosystems) at 95°C for 10 min, followed by 40 cycles of 95°C for 30 s and 57°C for 1 min, then 1 cycle of 98°C for 10 min, ending at 4°C.

To quantify miRNAs in EVs, 10 ng of EV-RNA were reversely transcribed using TaqMan TM microRNA Reverse Transcription Kit and miRNA specific RT primers (Cat. 4366597, Cat. 4427975, Applied Biosystems) in a final reaction volume of 20 μl. Then, 10 μl of cDNA (approximately 5 ng of cDNA) were added to a 2 × dPCR supermix for probe (Bio-Rad) and 1 μl 20 × TaqMan probe (Applied Biosystems) in a 20 μl reaction mix. Then, droplets were generated by loading the mix into a plastic cartridge with 70 μl of Droplet Generation Oil into the Droplet Generator (Bio-Rad Laboratories, Hercules, CA, USA). In addition, a no-template control was included in every assay. Droplets from each sample were carefully transferred to a 96-well PCR plate, and PCR amplification was carried out on a thermal cycler at 95°C for 10 min, then 40 cycles of 95°C for 15 s and 58°C for 1 min, and finally 98°C for 10 min and 4°C infinite holds. A ramping rate of 2°C/s was used in every step.

All the plates, for EV-miRNAs and HSPC-miRNAs, were read in the Droplet Reader (QX200 droplet digital PCR System, Bio-Rad) and analyzed using the QuantasoftTM version 1.7.4 software (Bio-Rad). Absolute quantification of each miRNA was calculated from the number of positive counts per panel using the Poisson distribution. miRNA quantification was reported as the number of copies/μl of PCR mixture and as copies for ng of EV-RNA (copies/ng of EV-RNA). All tests were performed in triplicates.

To identify the potential targets of miRNAs, the public available bioinformatics tool miRTargetLink Human was used (https://ccb-web.cs.uni-saarland.de/mirtargetlink/) (20).

### Statistical Analysis

Results were shown as mean ± SD of three independent experiments. Mann-Whitney U-test was used to analyze two-group comparisons. Cytofluorometric and cell cycle data were analyzed by two-way ANOVA followed by *post-hoc* multiple comparisons using Sidak's test. For all tests, a *p*-value ≤ 0.05 was taken as statistically significant.

## Results

### Multiple Myeloma Cell Line-Released EVs

**Figure 1:**
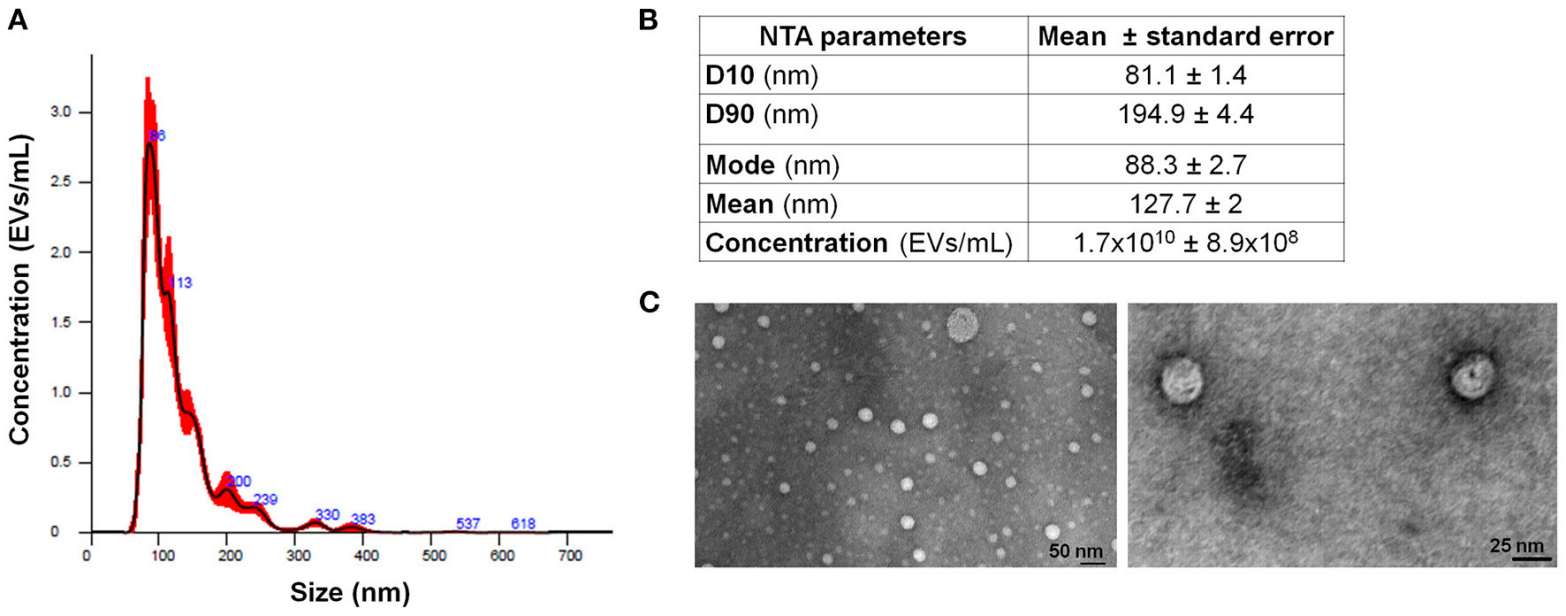
MM-EV characterization. **(A)** Representative histogram of hydrodynamic EV size distribution profile from RPMI 8226 cell line measured by NTA. **(B)** NTA data are expressed as D10, D90, mode, mean, and concentration of MM-derived EVs as mean value ± Standard Error. **(C)** Representative photos of MM cell line-derived EVs obtained by TEM (image magnification: 100 and 160 KX, respectively). The horizontal bar indicates 50 and 25 nm, respectively. MM, multiple myeloma; EVs, Extracellular vesicles; NTA, Nanoparticle Tracking Analysis.

Extracellular vesicles were isolated from the supernatant of MM cell line RPMI-8226 (MM-EVs). NTA was used to define both EV size and concentration. MM cells released a heterogeneous population of EVs in a diameter range of 50–400 nm, although most of them were very small in a range of 81–195 nm (D10 and D90), and the most abundant ones had a mode value of about 88 nm. Their concentration was about 1.7 × 10^10^/ml (Figures 1A,B).

Extracellular vesicles were qualitatively assessed by TEM showing round particles (Figure 1C). Based on the EV concentration and on the number of MM cells, we calculated that one million MM cells released about 135 × 10^6^ EVs.

### Multiple Myeloma-EVs Interacted With Normal HSPCs Modulating Their Viability and Differentiation

**Figure 2:**
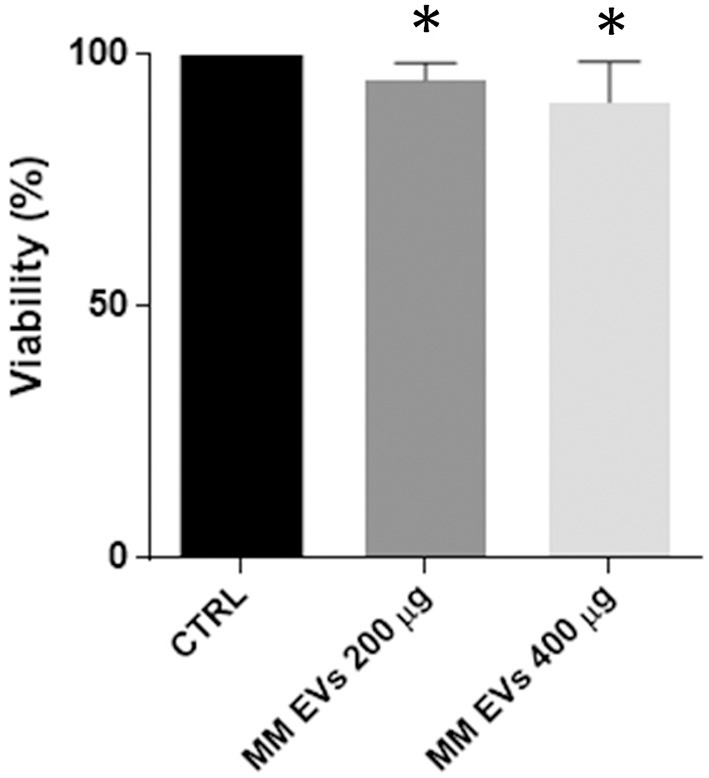
MM-EV effect on HSPC viability. HSPC viability after 20 h of treatment with 200 μg and 400 μg of MM-derived EVs. Results are expressed as the percent of cell viability normalized to control. The bar graphs represent the average with standard deviation from three independent experiments; ^*^ indicates *p* < 0.05. MM, multiple myeloma; EVs, Extracellular vesicles; HSPC, hematopoietic stem and progenitor cells.

Two doses of MM-EVs (200 and 400 μg) and PBS were added to 8.5 × 10^5^ HSPCs, and viability was analyzed after 20 h. MM-EVs induced a reduction of cell viability in a dose-dependent manner (*p* < 0.05; Figure 2).

**Figure 3:**
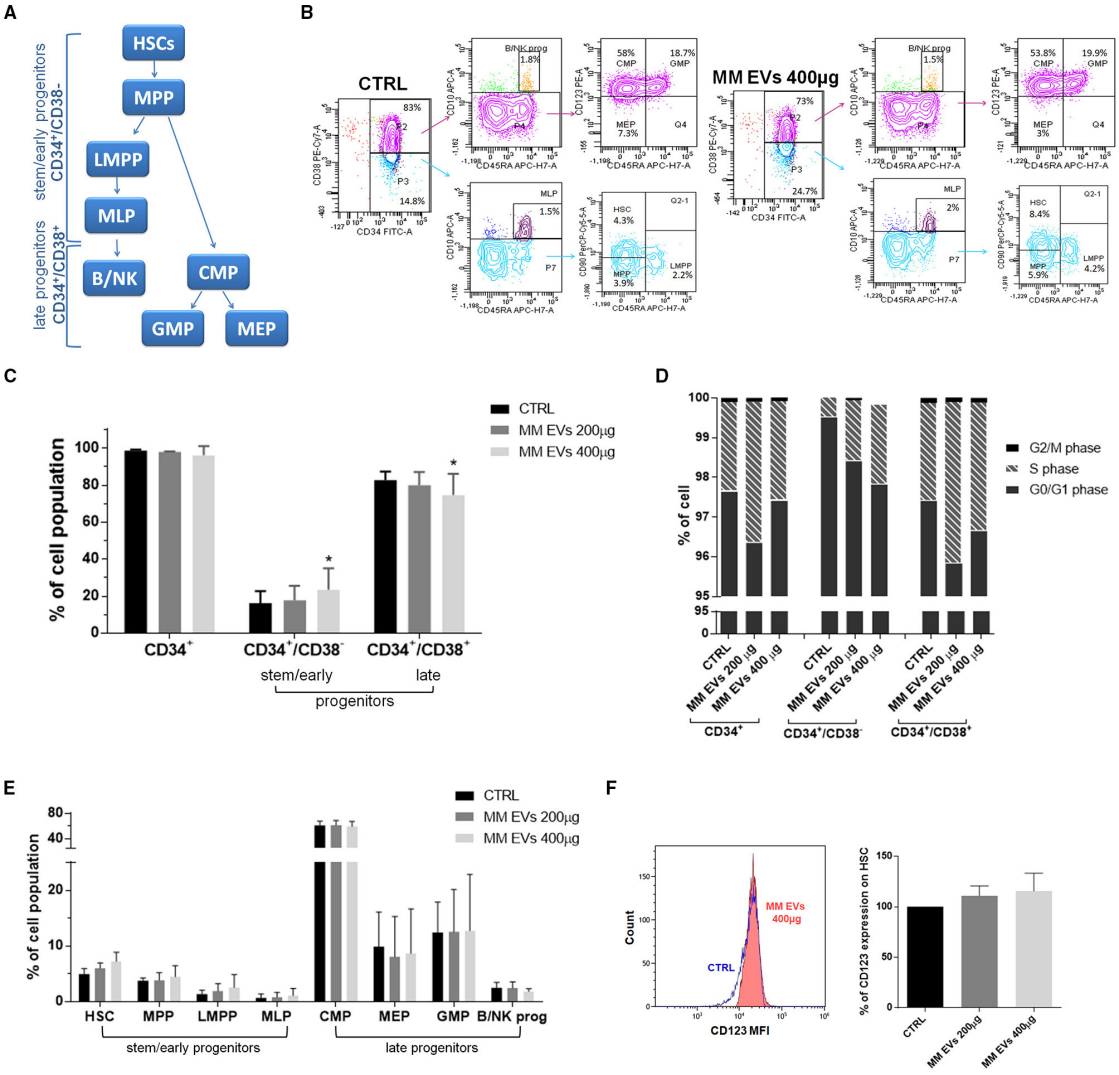
MM-EV effect on both HSPC cell cycle and differentiation and CD123 expression on HSCs. **(A)** Model of human lympho-myeloid differentiation. Stem/early progenitors include hematopoietic stem cells (HSCs), multipotent progenitors (MPP), lymphoid-primed multipotent progenitor (LMPP), multi-lymphoid progenitor (MLP); late progenitors include common myeloid progenitor (CMP), megakaryocyte erythroid progenitor (MEP), granulocyte macrophage progenitor (GMP), and B/NK progenitors (B/NK prog). Arrows indicate the derivation. **(B)** Representative cytofluorometric dot plots of populations in HSPCs treated or not with MM-EVs for 20 h. The cell percentage of each population was reported in dot plots. **(C)** Percentage of CD34^+^, stem/early, and late progenitors after 20 h of treatment with two doses of MM-EVs (MM-EVs 200 μg, MM-EVs 400 μg). **(D)** Percentage of cells in G0/G1, S, and G2/M phases on CD34^+^, CD34^+^/CD38^−^, and CD34^+^/CD38^+^ cells after treatment with two doses of MM-derived EVs. **(E)** Percentage of different HSPC populations after treatment with two doses of MM-EVs. **(F)** Percentage of CD123 expression on HSCs after treatment with two doses of MM-derived EVs. On the left representative histogram of CD123 mean fluorescence intensity (x-axis) in control (CTRL, blue) and treated HSPCs (MM-EVs 400 μg, red). The bar graphs represent the average with standard deviation from three independent experiments; ^*^ indicates *p* = 0.05. MM, multiple myeloma; EVs, Extracellular vesicles; HSPC, hematopoietic stem and progenitor cells.

To understand the downstream effects of MM-EVs on HPSCs, we examined the fate of specific HSPC subsets following MM-EV exposure using a multiparameter flow cytometer analysis with a combination of antibodies (CD34, CD38, CD45RA, CD10, CD90, and CD123) allowing us to distinguish the classical HSPC subsets (Figure 3A). Based on the absence/presence of differentiated lineage markers, we observed the expansion of stem/early progenitors (CD34^+^CD38^−−^ cells) accompanied by a reduction of late progenitors (CD34^+^CD38^+^ cells; *p* = 0.05; Figures 3B,C) in the MM-EV treated compared to control HPSCs. This effect was associated with an apparent cell cycle re-entry of progenitor cells associated with a reduction of the percentage of cells in the G0/G1 phase and an increase in the S and G2/M phases (*p* > 0.05; Figure 3D). In particular, the percentage of CD34^+^CD38^−−^ cells in the S phase was increased by 2.8- and 4.6-fold compared to untreated cells passing from first to second EV dose, while in CD34^+^CD38^+^ cells, this percentage was increased by 1.25- and 1.16-fold compared to control (Figure 3D).

More in detail, we detected an increase in the percentage of HSCs, multipotent progenitor (MPP), lymphoid-primed MPP (LMPPs), and multipotent lymphoid progenitors (MLPs), accompanied by a reduction of megakaryocyte erythroid progenitors (MEPs) and B and NK progenitors and by a slight increase of granulocyte macrophage progenitors (GMPs). No variations were observed for common myeloid progenitors (CMPs; *p* > 0.05; Figure 3E). The expression levels of specific differentiation markers, reported as mean fluorescence intensity (MFI), have not changed between control and treated HSPCs (Supplementary Table 1).

Interestingly, CD123, a well-characterized “leukemic” stem cell surface marker known to differentiate between phenotypically normal and “aberrant” hematopoietic stem cells, was increased in a dose-dependent fashion following treatment with MM-EVs (*p* = 0.07; Figure 3F).

### HSPC Colony-Forming Ability Was Impaired by MM-EVs

**Figure 4:**
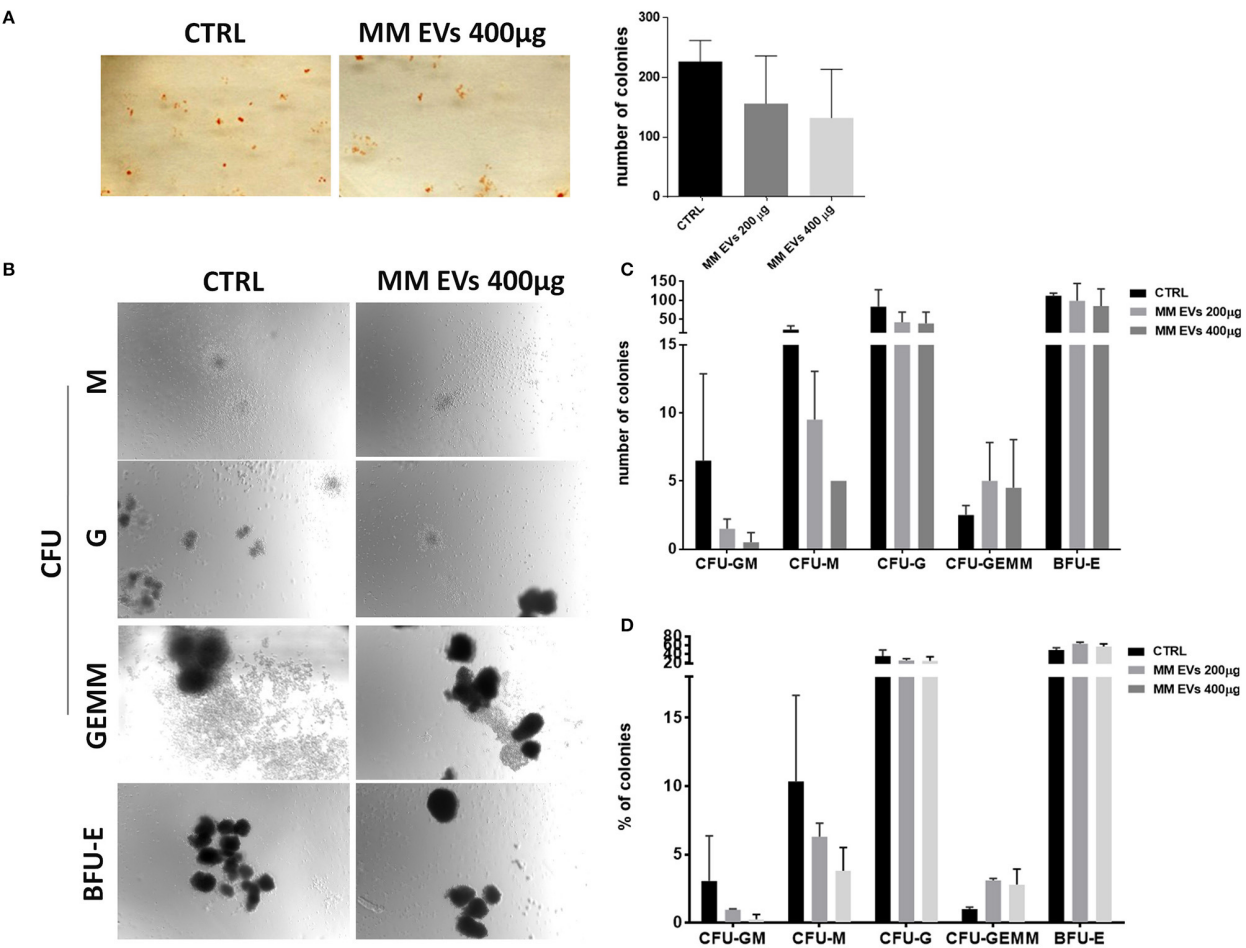
MM-EV effect on HSPC colony formation. **(A)** Representative photos of colony-forming unit (CFU) assay for HSPCs after 20 h of treatment with MM-EVs 400 μg (left) and means of the total number of colonies after treatment with two doses of MM-derived EVs (MM-EVs 200 μg, MM-EVs 400 μg; right); **(B)** representative photos of CFU-Granulocyte, Macrophage (CFU-GM), CFU Macrophage (CFU-M), CFU Granulocyte (CFU-G), CFU Granulocyte, Erythrocyte, Macrophage, Megakaryocyte (CFU-GEMM), and Burst-Forming-Unit Erythrocyte (BFU-E) after treatment with MM-EVs 400 μg; means of the total number **(C)** and proportion **(D)** of all type of colony after treatment with two doses of MM-derived EVs. The bar graphs represent the average with SD from three independent experiments. MM, multiple myeloma; EVs, Extracellular vesicles; HSPC, hematopoietic stem and progenitor cells.

To better characterize MM-EV effects, we performed methylcellulose CFU assays to determine whether MM-derived EVs could functionally affect the colony-forming ability of HSPCs. We observed that MM-EV-treated HSPCs gave rise to a reduced number of colonies compared to control cells (*p* > 0.05; Figure 4A). In particular, we reported a reduced number of CFU-GM/M/G and BFU-E, accompanied by an increase of CFU-GEMM in MM-EV treated compared to untreated cells (*p* > 0.05; Figures 4B,C). Analyzing the differences in percentage, CFU-GM/M/G were 3-, 1.5-, 1.5-fold decreased, respectively; while CFU-GEMM were 3-fold increased in MM-EV treated compared to untreated colonies (*p* > 0.05; Figure 4D).

### Multiple Myeloma-EVs Modulated CXCR4 Expression in HSPCs

**Figure 5:**
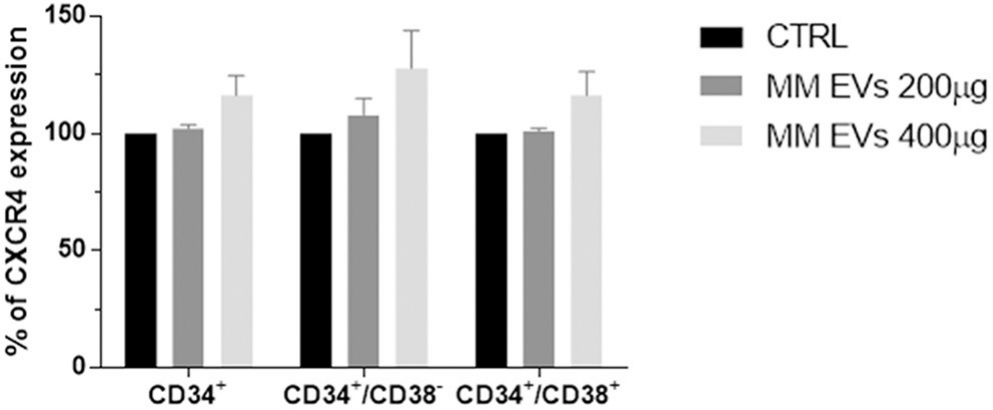
MM-EV effect on CXCR4 expression on HSPCs. Percentage of CXCR4 expression on CD34^+^, CD34^+^/CD38^−^, and CD34^+^/CD38^+^ cells after 20 h of treatment with two doses of MM-derived EVs (MM-EVs 200 μg, MM-EVs 400 μg). The bar graphs represent the average with standard deviation from three independent experiments. MM, multiple myeloma; EVs, Extracellular vesicles; HSPC, hematopoietic stem and progenitor cells.

Since the SDF-1/CXCR4 axis plays an important role in the physiology of HPSCs, we evaluated CXCR4 expression in HSPCs after MM-EV treatment observing an increased surface expression of CXCR4 in MM-EV-treated cells, in both stem/early and late progenitors with a major impact on stem/early progenitors at the higher EV dose (*p* > 0.05; Figure 5).

### Multiple Myeloma-EVs Dysregulated microRNAs in HPSCs

**Figure 6:**
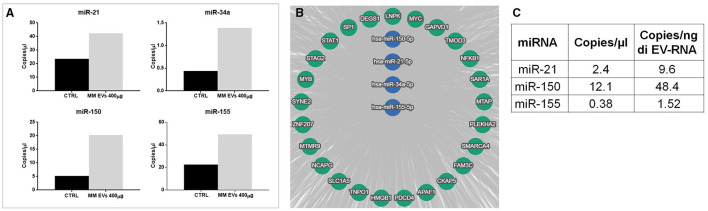
miRNA alteration in HSPCs after MM-EV interaction. **(A)** Digital PCR analysis for miR-21, miR-34a, miR-150, and miR-155 on HSPCs treated and not with 400 μg MM-derived EVs (the higher dose). **(B)** The target genes of hsa-miRNAs were analyzed by the bioinformatics tool miRTargetLink human. The central node represents miR-21, miR-34a, miR-150, and miR-155, and it was surrounded by the validated target with strong evidence. The bar graphs represent the average of three replicates. **(C)** The table showed miR-21, miR-150, and miR-155 amount as copies/μl of dPCR reaction mix and copies/ng of EV-RNA in EVs. MM, multiple myeloma; EVs, Extracellular vesicles; HSPC, hematopoietic stem and progenitor cells.

To understand the mechanisms responsible for the observed effects, we choose a series of miRNAs, which are important in stem cells and are generally considered dysregulated in the “transformation” of HSPCs. In particular, miR-21, miR-34, miR-150, and miR-155 were quantified in HPSCs after EV treatment. Specifically, digital PCR revealed a higher copy number of all analyzed miRNAs in HSPCs treated with MM-EVs compared to untreated cells (*p* > 0.05; Figure 6A). In particular, 42.3 and 49.3 copies of miR-21 and miR-155 in the treated group compared to 23.6 and 22.5 copies in untreated one were found, respectively. MiR-34a and miR-150 were about 3- and 4-fold over-expressed in the treated vs. control group, respectively.

We next investigated the potential targets of selected microRNAs by bioinformatics tool miRTargetLink Human. In Figure 6B, the central node represents the analyzed miRNAs, surrounded by their validated targets with strong evidence. Among them, we found the transcription factors SP1, STAT1 NFKB1, MYB largely described as a modulator of HSPC differentiation, self-renewal, and also of malignant transformation. All information about miR-targets is reported in Supplementary Table 2.

To demonstrate that EVs carried, and likely unloaded, microRNAs directly in HSPCs, the presence of miRNAs 21, 150, and 155, which were found to increase in HSPCs after treatment, was investigated in MM-EVs. Interestingly, all miRNAs were present, although with different levels, in MM-EVs (Figure 6C).

## Discussion

Multiple myeloma is characterized by hematopoietic suppression accompanied, at diagnosis, by anemia as a prevalent clinical symptom (21). The underlying mechanisms of MM-related anemia are incompletely understood. We hypothesized that hematopoiesis compromission is not only due to “a physical displacement” of HSPCs by BM MM-PC proliferation but also due to HPSC differentiation impairment. In particular, we hypothesized, and demonstrated here, for the first time, that MM-PCs interact with healthy HPSCs through the release of EVs, transferring miRNAs (and other information) and “transforming” them in terms of viability, cell cycle, phenotype, functions, and miRNoma. It is well known that MM-PCs, through EVs, are able to modify the BM microenvironment, interacting and modifying different BM cells (i.e., mesenchymal stem/stromal cells, endothelial cells, OBs/OCs, and immune cells), transforming them into a tumor-supporting environment (16, 22–24). To date, no data are available about the EV-mediated communication between MM-PCs and HSPCs.

In this study, as a source of MM-EVs, we used the MM cell line RPMI8226 demonstrating that they release small and medium EVs with a dimension's range of 40–500 nm and specific MM-PC surface markers, such as CD38 and CD138 (data not shown) (18). As recently reported, this is important in the clinical setting where, as we and others demonstrated, it is possible to monitor MM malignancy by analyzing these specific circulating EVs by a non-invasive peripheral blood sample, (18, 25, 26). We calculated that 1 × 10^6^ MM cells released a very large amount of EVs (135 × 10^6^). Of note, this amount of particles for cells could be underestimated because the NTA is unable to measure EVs smaller than 50 nm.

As a source of CD34^+^ cells, UCBs were used as reported by others (27, 28). The percentage of these cells in UCBs is very low, around 0.1–0.5%, so many bags (~40 bags) were needed to purify CD34^+^ cells enough for our experiments.

For functional experiments, we treated HSPCs with two doses of EVs based on protein quantification (and not on particle number). Interestingly, the EV amount used (20 and 40 μg to treat 1 × 10^5^ HSPCs) was in line with that used in another context such as that of AML-EVs and HSPCs where it was defined as a very small amount to induce effects in cells (29).

The HSPC pool distribution after MM-EV treatment was analyzed according to the classical differentiation model (Figure 3A) where it is possible to define two HSPC populations: stem/early progenitors (CD34^+^CD45RA^−−/+^CD38^−−^) and late progenitors (CD34^+^CD45RA^−−/+^CD38^+^; Figure 3A) (30, 31). The first ones are all multipotent but differ in their self-renewal ability, are capable of long-term engraftment (30, 32); while the late progenitors have limited self-renewal capacity and are described as giving rise to myeloid and lymphoid cells (30, 32, 33). After treatment with MM-EVs, an EV dose-dependent slight reduction of both cell number and CD34 percentage was observed. Unexpectedly, this reduction occurred with a significant increase of all stem/early progenitors accompanied by a reduction of late progenitors associated with a slight increase of GMP. This apparent discrepancy could be explained by different percentages of precursors on total CD34^+^ cells: the stem/early progenitors were ~15% while the late progenitors were ~85% (30, 32). Therefore, on total CD34^+^ cells the impact of late precursors dominates and covers that of stem/early ones. Interestingly, our data could explain what was reported in BM of patients with MM: the percentage of HSPCs was reduced, late precursors, such as MEP, was decreased, and the proportion of GMP was increased as compared to healthy donors (6, 34).

Of note, MM-EV treatment induced a reduction of B and NK progenitors in HSPCs and it is in line with the fact that also lymphoid differentiation is impaired.

In addition, in our setting, HSPCs passed from the non-replicative (G0/G1) to the replicative (S) phase as the result of their interaction with MM-EVs, and this effect was more pronounced in stem/early compared to late progenitors. This is very interesting because the stem/early progenitors were found to reside in a specific niche environment in the BM where they exist predominantly in a non-replicative and quiescent state (35) while, our data seems to say that MM-EVs break the balance between self-renewal and differentiation in favor of cell division.

In accordance with what has been observed on differentiation, HPSCs after interaction with MM-EVs reduced their ability to form colonies, especially BFU-E CFU-G/GM/M and, interestingly, again, this is in agreement with what has been reported for CD34 in patients with MM (6, 34).

With regards to the CXCR4 receptor, it is well known that it is a pivotal mediator of engraftment, retention, and multilineage differentiation of HSPCs in various SDF-1-expressing BM niches by regulating their migration, survival, and quiescence (36). In MM, CXCR4 is expressed also on malignant cells and SDF-1 levels are higher in the BM of patients compared to healthy individuals (37, 38). Of note, an increase of CXCR4 expression was observed in MM-EV-treated HSPCs. Similarly, on another cell population, such as OCs, MM-EVs increased CXCR4 expression contributing to their migration and promoting their differentiation (39). We could speculate that MM-PCs, by directly acting on HSPCs, could induce a BM HSCP-retention limiting the differentiation of HSPCs. Further studies are warranted to confirm this hypothesis.

We directly evaluated the influence of miRNAs on cell fate decisions in bulk human CD34^+^ cells following exposure to MM-EVs. We focused our attention on miR-21, miR-34a, miR-150, and miR-155, since they are the important mediators in hematopoietic stem cell differentiation and proliferation and, also, in the “malignancy-like transformation” of HSPCs (29, 40–44). Lu et al. (45) examined human BW-derived MEPs to identify miRNAs that influence cell fate choice between megakaryocytes or erythrocytes with the identification of miR-150 as crucial in this process.

A trend of overexpression of these miRNAs in HSPCs after EV contact was uncovered indicating that MM-derived EVs induced a miRNA alteration in HSPCs. It is already well known that EVs contain several species of non-coding RNAs, such as miRNAs, which can be transferred to target cells affecting their phenotypes, such as MSCs, fibroblasts, and endothelial cells (46, 47).

Interestingly, Raimondo et al. (15) recently, reported that the miRNA profile of MM cell line-derived EVs showed that miRNAs selected by us were abundant in these EVs.

We hypothesized, and demonstrated here, that miR-21, miR-34a, miR-150, and miR-155 packaged in EVs are transferred in recipient cells. Once in HSPCs, miRNAs can affect cell phenotype by targeting different transcripts. Indeed, bioinformatics analysis revealed that strong targets of these miRNAs, such as SF1, STAT1, NFKB1, and MYB, are involved in cell remodeling pathways, engraftment, differentiation, and self-renewal (42, 48). For example, in AML, miR-150- and miR-155-in EVs mediated suppression of c-Myb (49). Bianchi et al. (50) found miR-34a-5p upregulation in primary myelofibrosis CD34^+^ hematopoietic progenitor cells, demonstrating that its overexpression favors the megakaryocyte and monocyte commitment of CD34^+^ cells.

Many studies have explored the expression of CD123 in HSPCs, reporting its expression on most human CD34^+^ hematopoietic progenitors and its progressive loss during erythroid and megakaryocytic differentiation (51). Importantly, CD123 is expressed on leukemic stem cells and more differentiated leukemic blasts; this makes CD123 an attractive therapeutic target. Various agents have been developed as drugs able to target CD123 on malignant leukemic cells (52). In patients with MM who developed AML/Myelodysplastic Syndromes, stem, and progenitor cells (collected years before the onset of secondary disease) expressed high CD123 levels (53). Here we reported that the expression of CD123 was higher on MM-EV-treated HSCs compared to untreated ones. Based on our preliminary data on miRs and CD123, we speculated that MM-EVs could modify the original stem cells bringing to a “leukemic like” phenotype. Indeed, the transfer of EV-associated miR-21 and miR-29 is responsible for the expansion and the “transformation” of HSPCs in AML (42, 43).

We are very confident that the results obtained with MM-EVs are specific to Myeloma. In fact, we performed similar experiments using HSPCs and EVs extracted from acute myeloid leukemia cells and obtained different results in terms of differentiation and other functional assays (data not shown).

Of note, our study leads to an intriguing consideration. MM leads to functional impairment and diminution of all HSPC subsets with an emphasis on early erythroid precursors. These effects are apparently transient and largely dependent on the MM-related BM microenvironment (6). In fact, transplantation of HSPCs derived from MM patients into the BM of MM-free NOG mice showed enhanced engraftment and normal differentiation capacities (8). More importantly, the clinical data confirm the rapid and sustained engraftment in the majority of patients with MM after the elimination of MM cells by high-dose therapy (8). Indeed, this “transient” effect on HSPCs in MM could be explained in this way: the elimination of MM-PCs by therapy leads to failure of EV release and the consequent disappearance of their effects on HSPCs.

The present study supported our idea that EVs derived from tumor cells mediate two fundamental processes for the development/maintenance of malignancy: (1) inhibit normal hematopoiesis and (2) force normal cells toward a tumor phenotype.

It is clear that our results have the limit of the valuation of EVs from only one MM cell line, but we strongly believe that it represents the first and intriguing proof of concept of EV-mediated influence of malignant MM-PCs on normal HSPCs.

## Conclusions

**Figure 7:**
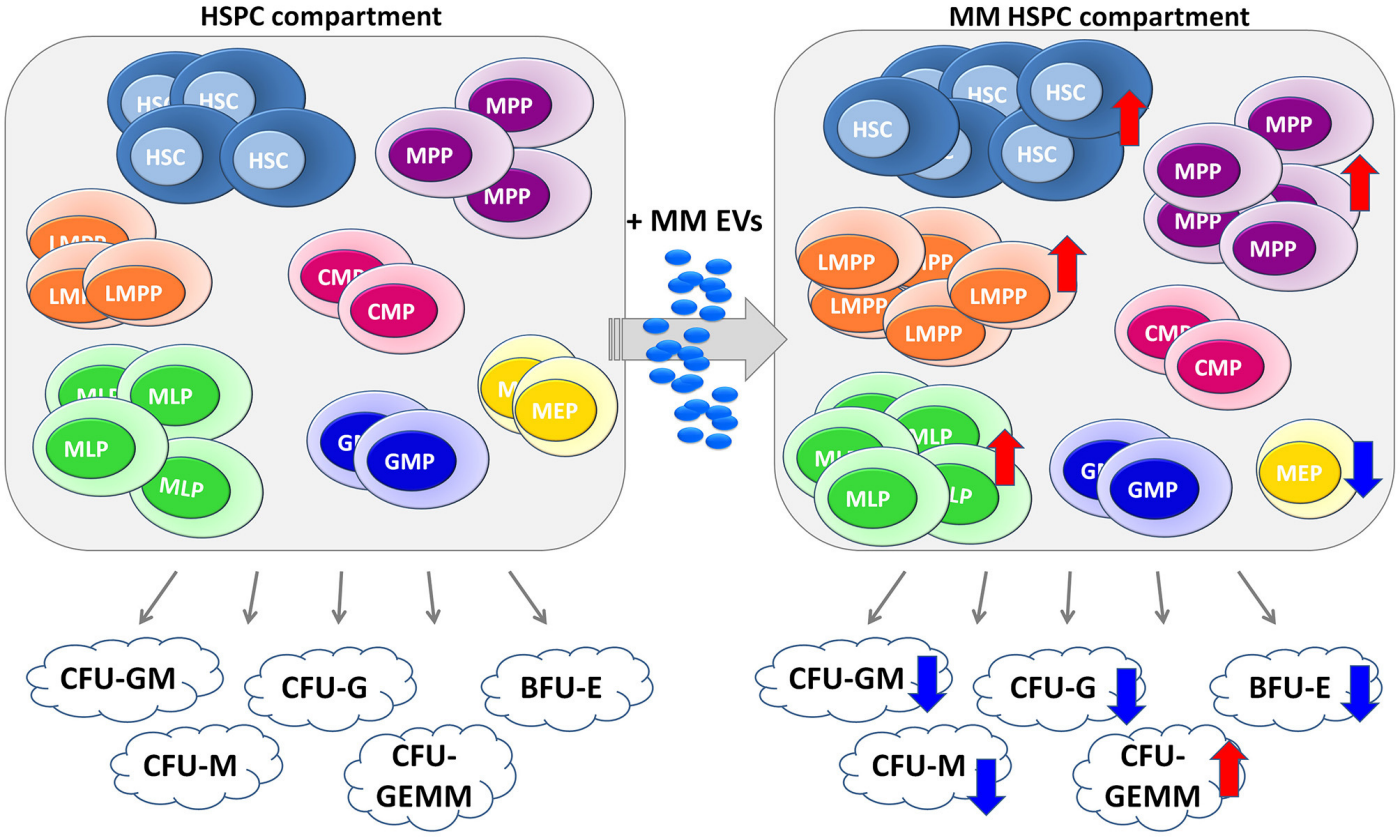
Cartoon of MM-EV action in HSPC compartment. The healthy HSPC compartment is characterized by different populations, such as hematopoietic stem cells (HSCs), multipotent progenitors (MPP), lymphoid-primed multipotent progenitor (LMPP), multi-lymphoid progenitor (MLP), common myeloid progenitor (CMP), megakaryocyte erythroid progenitor (MEP), granulocyte macrophage progenitor (GMP), and B/NK progenitors (B/NK prog). These progenitors are able to generate different colonies, such as CFU-Granulocyte, Macrophage (CFU-GM), CFU Macrophage (CFU-M), CFU Granulocyte (CFU-G), CFU Granulocyte, Erythrocyte, Macrophage, Megakaryocyte (CFU-GEMM), and Burst-Forming-Unit Erythrocyte (BFU-E, on the left). On the right, the EVs released by MM cells are captured by HSPCs, which in the response block their differentiation with consequent impairment of the ability to form colonies. The arrows indicate an increase (↑) or a decrease (↓) induced by MM-EVs on both different HSPC populations and colonies. MM, multiple myeloma; EVs, Extracellular vesicles; HSPC, hematopoietic stem and progenitor cells.

Taken together, our preliminary data suggest that hematopoietic impairment in MM could emerge from HSPCs as the result of the action of EVs released by MM-PCs (Figure 7). Strategies to block EV production and secretion and EV-induced reprogramming could represent novel exciting therapeutic approaches in MM.

## Data Availability Statement

The original contributions presented in the study are included in the article/Supplementary Material, further inquiries can be directed to the corresponding author.

## Ethics Statement

This study was approved by Ethics Committee of Scientific Institute for Research, Hospitalization and Healthcare (IRCCS)-CROB and that of Research Institute “Casa Sollievo della Sofferenza.” The patients/participants provided their written informed consent to participate in this study.

## Author Contributions

AC and LD designed the study. IL, ST, and DL constituted the project team for isolating both MM-EVs and CD34^+^ cells and for MM-EVs/CD34^+^ experiments. IL and DL isolated extracellular vesicles (EVs), performed both NTA, and flow cytometry analysis. AD performed TEM analysis of EVs. MS recruited UCB bags. ST conducted the digital PCR for miRNAs. AC, LD, and AS provided a critical review of the data analysis. AC wrote the first and final manuscripts. All authors approved the final version of the manuscript.

## Funding

This research was supported by Ricerca Corrente 2019-2020 of IRCCS-CROB by the Italian Ministry of Health.

## Conflict of Interest

The authors declare that the research was conducted in the absence of any commercial or financial relationships that could be construed as a potential conflict of interest.

## Publisher's Note

All claims expressed in this article are solely those of the authors and do not necessarily represent those of their affiliated organizations, or those of the publisher, the editors and the reviewers. Any product that may be evaluated in this article, or claim that may be made by its manufacturer, is not guaranteed or endorsed by the publisher.
