# Supplementary material for: Multiple Myeloma-Derived Extracellular Vesicles Impair Normal Hematopoiesis by Acting on Hematopoietic Stem and Progenitor Cells
Source: Front Med (Lausanne). 2021 Dec 16;8:793040. doi: 10.3389/fmed.2021.793040 (PMC8716627; doi:10.3389/fmed.2021.793040)
Supplement: Supplementary file 1 [file Table_1.DOCX]

| **Supplementary Table 1:** Mean fluorescence intensity values of cell surface markers for each HSPC populations | | | |
| --- | --- | --- | --- |
| **Cell surface marker** | **Population** | **CTRL** | **+ MM-EVs** |
| **CD34** | CD34^+^38^-^ | 25.23 | 24.83 |
|  | CD34^+^38^+^ | 26.80 | 26.86 |
| **CD38** | CD34^+^38^-^ | 1.31 | 1.35 |
|  | CD34^+^38^+^ | 5.46 | 6.00 |
| **CD90** | HSC | 14.21 | 12.88 |
|  | MPP | 6.93 | 7.48 |
|  | LMPP | 10.40 | 8.52 |
| **CD10** | MLP | 11.59 | 9.91 |
| **CD123** | CMP | 20.64 | 20.43 |
|  | MEP | 7.66 | 7.71 |
|  | GMP | 25.87 | 25.11 |
| **CD10** | B/NK prog | 18.63 | 19.32 |
| CTRL = Not treated HSPCs  + MM-EVs = HSPCs treated with 400ug of MM-EVs | | | |
